# *catena*-Poly[bis­(μ_4_-adipato-1:2:1′:2′κ^4^ *O* ^1^:*O* ^1′^:*O* ^4^:*O* ^4′^)bis­(*N*,*N*-dimethyl­formamide)-1κ*O*,2κ*O*-dicopper(II)]

**DOI:** 10.1107/S1600536810036093

**Published:** 2010-09-15

**Authors:** Guo-Yun Wu, Seik Weng Ng

**Affiliations:** a Hunan Medical Technical Secondary School, Changsha, Hunan 410014, People’s Republic of China; b Department of Chemistry, University of Malaya, 50603 Kuala Lumpur, Malaysia

## Abstract

In the title polymeric complex, [Cu_2_(C_6_H_8_O_4_)_2_(C_3_H_7_NO)_2_]_*n*_, the carboxyl­ate groups of the approximately *U*-shaped adipate dianion each bridge a pair of inversion-related, DMF-coordinated copper(II) atoms, generating a ribbon motif that runs along the *b* axis. The geometry of the copper(II) atom is distorted square-pyramidal; the apical site is occupied by the O atom of the DMF mol­ecule whereas the four basal sites are occupied by carboxyl­ate O atoms.

## Related literature

For the crystal structure of diaquaadipatocopper(II), see: Bakalbassis *et al.* (2001 ▶); Zheng *et al.* (2001 ▶).
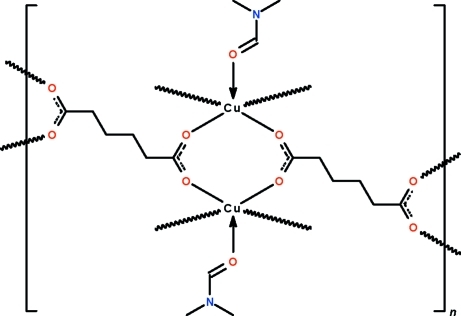


## Experimental

### 

#### Crystal data

[Cu_2_(C_6_H_8_O_4_)_2_(C_3_H_7_NO)_2_]
*M* *_r_* = 561.52
Monoclinic, 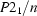

*a* = 9.4764 (5) Å
*b* = 8.2618 (5) Å
*c* = 15.0990 (8) Å
β = 106.259 (1)°
*V* = 1134.85 (11) Å^3^
*Z* = 2
Mo *K*α radiation
μ = 1.93 mm^−1^
*T* = 173 K
0.45 × 0.40 × 0.15 mm

#### Data collection

Bruker SMART APEX diffractometer
Absorption correction: multi-scan (*SADABS*; Sheldrick, 1996 ▶) *T*_min_ = 0.477, *T*_max_ = 0.761
5917 measured reflections
2428 independent reflections
2153 reflections with *I* > 2σ(*I*)
*R*_int_ = 0.018

#### Refinement

*R*[*F* ^2^ > 2σ(*F* ^2^)] = 0.024
*wR*(*F* ^2^) = 0.072
*S* = 1.03
2428 reflections
147 parameters
H-atom parameters constrained
Δρ_max_ = 0.41 e Å^−3^
Δρ_min_ = −0.23 e Å^−3^

### 

Data collection: *APEX2* (Bruker, 2004 ▶); cell refinement: *SAINT* (Bruker, 2004 ▶); data reduction: *SAINT*; program(s) used to solve structure: *SHELXS97* (Sheldrick, 2008 ▶); program(s) used to refine structure: *SHELXL97* (Sheldrick, 2008 ▶); molecular graphics: *X-SEED* (Barbour, 2001 ▶); software used to prepare material for publication: *publCIF* (Westrip, 2010 ▶).

## Supplementary Material

Crystal structure: contains datablocks global, I. DOI: 10.1107/S1600536810036093/xu5027sup1.cif

Structure factors: contains datablocks I. DOI: 10.1107/S1600536810036093/xu5027Isup2.hkl

Additional supplementary materials:  crystallographic information; 3D view; checkCIF report

## Figures and Tables

**Table 1:** Selected bond lengths (Å)

Cu1—O1	1.9683 (14)
Cu1—O2^i^	1.9716 (14)
Cu1—O3^ii^	1.9695 (13)
Cu1—O4^iii^	1.9584 (14)
Cu1—O5	2.1646 (15)

Symmetry codes: (i) 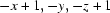; (ii) 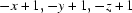; (iii) 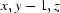.

**Fig. 1.**
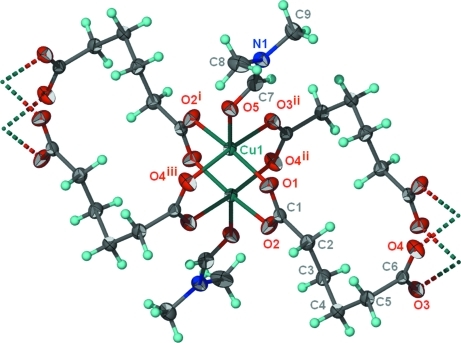
Thermal ellipsoid plot (Barbour, 2001) of a portion of the ribbon structure of Cu2(C6H8O4)2(C3H7NO)2 at the 70% probability level; hydrogen atoms are drawn as spheres of arbitrary radius.

## supplementary crystallographic information

### Comment

Copper adipate furnishes a number of adducts with oxygen- and nitrogen-donor ligands. The parent compound itself exists as dihydrate, with the copper atom in a square-planar environment (Bakalbassis *et al.*, 2001; Zheng *et al.*, 2001). The four-coordinate nature explains the ability of the compound to expand the coordination number of the metal atom. In the present study, the DMF solvent used in the synthesis functions as donor ligand. The DMF adduct is formally the dicopper diadipate bis-adduct (Scheme I). The carboxyl –CO_2_ ends of the approximately *U*-shape adipate dianion of polymeric Cu_2_(C_6_H_8_O_4_)_2_(C_3_H_7_NO)_2_ each bridges a pair of inversion-related, DMF-coordinated copper atoms (Fig. 1) to generate a ribbon motif that runs along the *b*-axis of the monoclinic unit cell. The geometry of the copper atom is a square pyramid; the apical site is occupied by the O atom of the DMF molecule whereas the four basal sites are occupied by the O atoms of the carboxyl ends.

### Experimental

(1*H*-Benzimidazol-2-yl)-methanol (0.074 g, 0.5 mmol) was dissolved in a methanol/DMF mixture (*v*/*v* 1:1, 20 ml) and to this was added copper nitrate trihydrate (0.241 g, 1 mmol) followed by adipic acid (0.073 g, 0.5 mmol). The mixture was filtered and then set aside. Blue crystals were isolated after two weeks.

### Refinement

Carbon-bound H-atoms were placed in calculated positions (C—H 0.95 to 0.98 Å) and were included in the refinement in the riding model approximation, with *U*(H) set to 1.2 to 1.5*U*(C).

### Crystal data

**Table:**

[Cu_2_(C_6_H_8_O_4_)_2_(C_3_H_7_NO)_2_]	*F*(000) = 580
*M**_r_* = 561.52	*D*_x_ = 1.643 Mg m^−^^3^
Monoclinic, *P*2_1_/*n*	Mo *K*α radiation, λ = 0.71073 Å
Hall symbol: -P 2yn	Cell parameters from 4192 reflections
*a* = 9.4764 (5) Å	θ = 2.3–27.0°
*b* = 8.2618 (5) Å	µ = 1.93 mm^−^^1^
*c* = 15.0990 (8) Å	*T* = 173 K
β = 106.259 (1)°	Block, blue
*V* = 1134.85 (11) Å^3^	0.45 × 0.40 × 0.15 mm
*Z* = 2	

### Data collection

**Table:**

Bruker SMART APEX diffractometer	2428 independent reflections
Radiation source: fine-focus sealed tube	2153 reflections with *I* > 2σ(*I*)
graphite	*R*_int_ = 0.018
ω scans	θ_max_ = 27.0°, θ_min_ = 2.3°
Absorption correction: multi-scan (*SADABS*; Sheldrick, 1996)	*h* = −9→12
*T*_min_ = 0.477, *T*_max_ = 0.761	*k* = −10→10
5917 measured reflections	*l* = −19→12

### Refinement

**Table:**

Refinement on *F*^2^	Primary atom site location: structure-invariant direct methods
Least-squares matrix: full	Secondary atom site location: difference Fourier map
*R*[*F*^2^ > 2σ(*F*^2^)] = 0.024	Hydrogen site location: inferred from neighbouring sites
*wR*(*F*^2^) = 0.072	H-atom parameters constrained
*S* = 1.03	*w* = 1/[σ^2^(*F*_o_^2^) + (0.0347*P*)^2^ + 1.1165*P*] where *P* = (*F*_o_^2^ + 2*F*_c_^2^)/3
2428 reflections	(Δ/σ)_max_ = 0.001
147 parameters	Δρ_max_ = 0.41 e Å^−^^3^
0 restraints	Δρ_min_ = −0.23 e Å^−^^3^

### Fractional atomic coordinates and isotropic or equivalent isotropic displacement parameters (Å^2^)

**Table:**

	*x*	*y*	*z*	*U*_iso_*/*U*_eq_	
Cu1	0.43225 (2)	−0.00484 (2)	0.564117 (15)	0.01597 (9)	
O1	0.52755 (16)	0.20568 (17)	0.60153 (10)	0.0269 (3)	
O2	0.64565 (17)	0.21315 (17)	0.49265 (10)	0.0266 (3)	
O3	0.72585 (15)	0.89246 (17)	0.52815 (9)	0.0242 (3)	
O4	0.60808 (16)	0.88250 (19)	0.63743 (10)	0.0282 (3)	
O5	0.31493 (16)	−0.01217 (17)	0.66816 (10)	0.0240 (3)	
N1	0.12143 (18)	0.0799 (2)	0.71426 (11)	0.0231 (3)	
C1	0.6147 (2)	0.2692 (2)	0.56190 (13)	0.0192 (4)	
C2	0.6893 (2)	0.4247 (2)	0.60485 (14)	0.0217 (4)	
H2A	0.7516	0.3998	0.6678	0.026*	
H2B	0.6124	0.5015	0.6110	0.026*	
C3	0.7844 (2)	0.5086 (2)	0.55190 (14)	0.0221 (4)	
H3A	0.7201	0.5715	0.5004	0.027*	
H3B	0.8351	0.4253	0.5249	0.027*	
C4	0.8997 (2)	0.6224 (2)	0.61229 (14)	0.0230 (4)	
H4A	0.9740	0.5566	0.6569	0.028*	
H4B	0.9504	0.6802	0.5726	0.028*	
C5	0.8366 (2)	0.7468 (2)	0.66538 (13)	0.0218 (4)	
H5A	0.7991	0.6896	0.7118	0.026*	
H5B	0.9167	0.8196	0.6990	0.026*	
C6	0.7139 (2)	0.8484 (2)	0.60538 (12)	0.0176 (4)	
C7	0.1952 (2)	0.0576 (2)	0.65288 (14)	0.0238 (4)	
H7	0.1530	0.0984	0.5924	0.029*	
C8	0.1795 (3)	0.0257 (3)	0.80898 (15)	0.0324 (5)	
H8A	0.2752	−0.0261	0.8166	0.049*	
H8B	0.1114	−0.0523	0.8236	0.049*	
H8C	0.1911	0.1187	0.8506	0.049*	
C9	−0.0149 (2)	0.1724 (3)	0.69183 (16)	0.0311 (5)	
H9A	−0.0436	0.1998	0.6261	0.047*	
H9B	−0.0003	0.2720	0.7285	0.047*	
H9C	−0.0925	0.1074	0.7058	0.047*	

### Atomic displacement parameters (Å^2^)

**Table:**

	*U*^11^	*U*^22^	*U*^33^	*U*^12^	*U*^13^	*U*^23^
Cu1	0.01828 (14)	0.01576 (14)	0.01519 (13)	0.00059 (8)	0.00683 (10)	−0.00014 (8)
O1	0.0302 (8)	0.0256 (7)	0.0286 (8)	−0.0089 (6)	0.0143 (6)	−0.0088 (6)
O2	0.0381 (8)	0.0200 (7)	0.0264 (7)	−0.0063 (6)	0.0169 (6)	−0.0056 (6)
O3	0.0232 (7)	0.0299 (7)	0.0204 (7)	0.0058 (6)	0.0075 (6)	0.0064 (6)
O4	0.0275 (8)	0.0383 (8)	0.0212 (7)	0.0110 (6)	0.0111 (6)	0.0085 (6)
O5	0.0225 (7)	0.0303 (8)	0.0215 (7)	0.0058 (6)	0.0100 (6)	0.0036 (5)
N1	0.0221 (8)	0.0269 (9)	0.0225 (8)	0.0027 (7)	0.0099 (7)	0.0002 (7)
C1	0.0186 (9)	0.0169 (8)	0.0206 (9)	0.0030 (7)	0.0032 (7)	0.0001 (7)
C2	0.0244 (10)	0.0186 (9)	0.0230 (9)	−0.0008 (8)	0.0082 (8)	−0.0039 (7)
C3	0.0262 (10)	0.0187 (9)	0.0227 (9)	−0.0004 (7)	0.0089 (8)	−0.0017 (7)
C4	0.0190 (9)	0.0205 (9)	0.0300 (10)	0.0017 (7)	0.0074 (8)	0.0024 (8)
C5	0.0215 (9)	0.0191 (9)	0.0214 (9)	−0.0002 (7)	0.0003 (8)	−0.0002 (7)
C6	0.0186 (9)	0.0144 (8)	0.0184 (9)	−0.0013 (7)	0.0030 (7)	−0.0015 (7)
C7	0.0282 (10)	0.0250 (10)	0.0213 (9)	0.0016 (8)	0.0122 (8)	0.0024 (8)
C8	0.0252 (11)	0.0518 (14)	0.0216 (10)	0.0005 (10)	0.0092 (9)	0.0013 (9)
C9	0.0284 (11)	0.0294 (11)	0.0387 (12)	0.0063 (9)	0.0150 (10)	−0.0017 (9)

### Geometric parameters (Å, °)

**Table:**

Cu1—O1	1.9683 (14)	C2—H2B	0.9900
Cu1—O2^i^	1.9716 (14)	C3—C4	1.533 (3)
Cu1—O3^ii^	1.9695 (13)	C3—H3A	0.9900
Cu1—O4^iii^	1.9584 (14)	C3—H3B	0.9900
Cu1—O5	2.1646 (15)	C4—C5	1.525 (3)
Cu1—Cu1^i^	2.6069 (4)	C4—H4A	0.9900
O1—C1	1.262 (2)	C4—H4B	0.9900
O2—C1	1.251 (2)	C5—C6	1.512 (3)
O3—C6	1.256 (2)	C5—H5A	0.9900
O4—C6	1.262 (2)	C5—H5B	0.9900
O5—C7	1.235 (3)	C7—H7	0.9500
N1—C7	1.321 (3)	C8—H8A	0.9800
N1—C8	1.452 (3)	C8—H8B	0.9800
N1—C9	1.457 (3)	C8—H8C	0.9800
C1—C2	1.521 (3)	C9—H9A	0.9800
C2—C3	1.529 (3)	C9—H9B	0.9800
C2—H2A	0.9900	C9—H9C	0.9800
			
O4^iii^—Cu1—O1	90.48 (7)	C4—C3—H3A	108.9
O4^iii^—Cu1—O3^ii^	169.19 (6)	C2—C3—H3B	108.9
O1—Cu1—O3^ii^	89.05 (6)	C4—C3—H3B	108.9
O4^iii^—Cu1—O2^i^	89.23 (7)	H3A—C3—H3B	107.8
O1—Cu1—O2^i^	169.11 (6)	C5—C4—C3	114.04 (16)
O3^ii^—Cu1—O2^i^	89.19 (6)	C5—C4—H4A	108.7
O4^iii^—Cu1—O5	96.03 (6)	C3—C4—H4A	108.7
O1—Cu1—O5	95.97 (6)	C5—C4—H4B	108.7
O3^ii^—Cu1—O5	94.76 (6)	C3—C4—H4B	108.7
O2^i^—Cu1—O5	94.88 (6)	H4A—C4—H4B	107.6
O4^iii^—Cu1—Cu1^i^	85.20 (4)	C6—C5—C4	114.07 (16)
O1—Cu1—Cu1^i^	84.51 (4)	C6—C5—H5A	108.7
O3^ii^—Cu1—Cu1^i^	84.00 (4)	C4—C5—H5A	108.7
O2^i^—Cu1—Cu1^i^	84.62 (4)	C6—C5—H5B	108.7
O5—Cu1—Cu1^i^	178.67 (4)	C4—C5—H5B	108.7
C1—O1—Cu1	122.68 (12)	H5A—C5—H5B	107.6
C1—O2—Cu1^i^	122.64 (13)	O3—C6—O4	125.38 (18)
C6—O3—Cu1^ii^	123.01 (13)	O3—C6—C5	117.60 (17)
C6—O4—Cu1^iv^	122.10 (12)	O4—C6—C5	117.02 (16)
C7—O5—Cu1	118.87 (13)	O5—C7—N1	124.96 (19)
C7—N1—C8	121.24 (17)	O5—C7—H7	117.5
C7—N1—C9	121.29 (18)	N1—C7—H7	117.5
C8—N1—C9	117.24 (17)	N1—C8—H8A	109.5
O2—C1—O1	125.47 (18)	N1—C8—H8B	109.5
O2—C1—C2	118.63 (17)	H8A—C8—H8B	109.5
O1—C1—C2	115.89 (16)	N1—C8—H8C	109.5
C1—C2—C3	115.59 (16)	H8A—C8—H8C	109.5
C1—C2—H2A	108.4	H8B—C8—H8C	109.5
C3—C2—H2A	108.4	N1—C9—H9A	109.5
C1—C2—H2B	108.4	N1—C9—H9B	109.5
C3—C2—H2B	108.4	H9A—C9—H9B	109.5
H2A—C2—H2B	107.4	N1—C9—H9C	109.5
C2—C3—C4	113.16 (16)	H9A—C9—H9C	109.5
C2—C3—H3A	108.9	H9B—C9—H9C	109.5
			
O4^iii^—Cu1—O1—C1	82.83 (16)	O1—C1—C2—C3	−175.00 (17)
O3^ii^—Cu1—O1—C1	−86.36 (16)	C1—C2—C3—C4	−158.25 (16)
O2^i^—Cu1—O1—C1	−5.6 (4)	C2—C3—C4—C5	−53.1 (2)
O5—Cu1—O1—C1	178.95 (15)	C3—C4—C5—C6	−54.7 (2)
Cu1^i^—Cu1—O1—C1	−2.30 (15)	Cu1^ii^—O3—C6—O4	−7.1 (3)
O4^iii^—Cu1—O5—C7	175.47 (15)	Cu1^ii^—O3—C6—C5	173.25 (12)
O1—Cu1—O5—C7	84.35 (16)	Cu1^iv^—O4—C6—O3	6.2 (3)
O3^ii^—Cu1—O5—C7	−5.19 (16)	Cu1^iv^—O4—C6—C5	−174.09 (12)
O2^i^—Cu1—O5—C7	−94.78 (16)	C4—C5—C6—O3	−38.7 (2)
Cu1^i^—O2—C1—O1	−2.9 (3)	C4—C5—C6—O4	141.63 (18)
Cu1^i^—O2—C1—C2	175.51 (12)	Cu1—O5—C7—N1	−171.07 (16)
Cu1—O1—C1—O2	3.8 (3)	C8—N1—C7—O5	1.7 (3)
Cu1—O1—C1—C2	−174.68 (12)	C9—N1—C7—O5	176.2 (2)
O2—C1—C2—C3	6.4 (3)		

Symmetry codes: (i) −*x*+1, −*y*, −*z*+1; (ii) −*x*+1, −*y*+1, −*z*+1; (iii) *x*, *y*−1, *z*; (iv) *x*, *y*+1, *z*.
